# Protective Enterotoxigenic *Escherichia coli* Antigens in a Murine Intranasal Challenge Model

**DOI:** 10.1371/journal.pntd.0003924

**Published:** 2015-08-05

**Authors:** Amit Kumar, Mike Hays, Francis Lim, Leonard J. Foster, Mingxu Zhou, Guoqiang Zhu, Tracy Miesner, Philip R. Hardwidge

**Affiliations:** 1 College of Veterinary Medicine, Kansas State University, Manhattan, Kansas, United States of America; 2 Department of Biochemistry & Molecular Biology and Centre for High-Throughput Biology, University of British Columbia, Vancouver, British Columbia, Canada; 3 College of Veterinary Medicine, Yangzhou University and Jiangsu Co-Innovation Center for Important Animal Infectious Diseases and Zoonoses, Yangzhou, Jiangsu, China; University of California San Diego School of Medicine, UNITED STATES

## Abstract

Enterotoxigenic *Escherichia coli* (ETEC) is an endemic health threat in underdeveloped nations. Despite the significant effort extended to vaccine trials using ETEC colonization factors, these approaches have generally not been especially effective in mediating cross-protective immunity. We used quantitative proteomics to identify 24 proteins that differed in abundance in membrane protein preparations derived from wild-type vs. a type II secretion system mutant of ETEC. We expressed and purified a subset of these proteins and identified nine antigens that generated significant immune responses in mice. Sera from mice immunized with either the MltA-interacting protein MipA, the periplasmic chaperone seventeen kilodalton protein, Skp, or a long-chain fatty acid outer membrane transporter, ETEC_2479, reduced the adherence of multiple ETEC strains differing in colonization factor expression to human intestinal epithelial cells. In intranasal challenge assays of mice, immunization with ETEC_2479 protected 88% of mice from an otherwise lethal challenge with ETEC H10407. Immunization with either Skp or MipA provided an intermediate degree of protection, 68 and 64%, respectively. Protection was significantly correlated with the induction of a secretory immunoglobulin A response. This study has identified several proteins that are conserved among heterologous ETEC strains and may thus potentially improve cross-protective efficacy if incorporated into future vaccine designs.

## Author Summary

Diarrheal disease is an endemic health threat in underdeveloped nations. One of the major causative agents of diarrheal disease is a group of bacteria collectively known as enterotoxigenic *Escherichia coli* (ETEC). These organisms can cause disease symptoms ranging from mild diarrhea to a more severe, cholera-like form. We were interested in characterizing ETEC proteins that can generate a protective immune response as the first step in identifying potential new vaccine candidates. We used proteomics to identify a subset of ETEC proteins and then characterized this subset for their ability to inhibit ETEC binding to cultured intestinal epithelial cells. We then vaccinated mice with the most promising antigen candidates and were able to identify three proteins that protected mice from clinical signs of disease normally caused by ETEC infection. We suggest that future characterization of these proteins may potentially improve our collective efforts to create safe, effective, and broadly protective ETEC vaccines.

## Introduction

Enterotoxigenic *Escherichia coli* (ETEC) is a significant cause of human morbidity due to infectious diarrhea and resultant malnutrition [1]. A recent Global Enteric Multicenter study conducted over a 3-year period to identify the etiology of pediatric diarrheal diseases in sub-Saharan Africa and South Asia found that ETEC infection led to moderate to severe diarrhea in 60–70% of ETEC infected patients and found that ETEC was present at all study sites [2].

ETEC are a diverse group of pathogens that colonize the small intestine, where they attach to mucosal surfaces using surface antigens known as colonization factors [CFs; [3]. ETEC infections are associated with an acute watery diarrhea that can lead to rapid dehydration [1]. At least 25 unique CFs have been identified [4]. ETEC strains also express heat-labile (LT) and/or heat-stable (ST) enterotoxins [5]. The enzymatic activities of these enterotoxins cause diarrhea by ultimately inducing water and electrolyte loss from the intestine of infected subjects [5].

Several strategies have been used for ETEC vaccine development. Purified CFs have been used as oral immunogens to provide protection against later challenge with ETEC expressing homologous CFs [6]. A commonly used approach has involved using the cholera toxin B subunit (CT-B) with formalin-inactivated ETEC strains expressing the most prevalent CFs [7]. This approach showed that the vaccine elicited IgA responses against the different CFs that were used [6]. However, further trials based on this approach with vaccines expressing CFA/I, CS1-3, CS5, and a recombinant CT-B suggested the need to improve vaccine safety in infants and young children [8,9]. A new version of this oral vaccine with an increased level of CF expression is being tested [10].

An approach with a live attenuated oral ETEC vaccine was also taken where an ETEC variant (E1392/75-2A) that had lost the capacity to produce toxin but still expressed CFA/II was used for oral vaccination. The vaccination showed 75% protection against ETEC expressing CFA/II [11]. E1392/75-2A was further attenuated and found to be immunogenic and safe to administer to humans [11]. However, challenge studies have, to our knowledge, not been conducted to determine protective efficacy. A recent study combined six ETEC vaccine strains expressing different CFs with the LT B subunit [12]. This vaccine formulation (ACE527), which was used in a phase I trial [12], was well tolerated and immunogenic [13,14] and may be the subject of future development.

An attenuated *S*. *flexneri* 2a vaccine strain CVD 1204, bearing deletions in the guanine nucleotide biosynthetic pathway (Δ*guaBA*), was used to express different ETEC colonization factors along with mutated forms of LT (LThK63 or LThR72); [15]). Following intranasal immunization of guinea pigs, both serum IgG and mucosal IgA responses against the CFs were detected. However, the immune response was not consistent against the mutant LT [16]. This approach was improved upon by using four different ETEC fimbrial antigens (CFA/I, CS2, CS3, and CS4) and LThk63 expressed separately in CVD 1204 [17]. However, the invasive capacity of *S*. *flexneri* Δ*guaBA* was significantly reduced, which may limit its ability to stimulate robust immune responses [18], and expression of ETEC CFs further reduces its invasiveness [19]. An invasive strain, *S*. *flexneri* 2a (SC608) was also developed for heterologous antigen expression [20]. All these studies showed a significant immune response against ETEC CFs. However, none of these immunization studies were, to our knowledge, followed with ETEC challenge, due to lack of a proper animal model to assess directly protection against ETEC infection.

Plasmid-based antigens such as EtpA and EatA have protective efficacy against ETEC challenge in a mouse model [4]. EtpA is secreted by a two-partner secretion system and functions as an ETEC adhesin that promotes colonization of mice [21]. Immunization with recombinant EtpA provided protection against ETEC H10407 challenge, based upon reduced ETEC colonization of the mouse intestine [21]. EatA is a serine protease that degrades EtpA [22]. EatA also degrades MUC2, a major component of intestinal mucin [23]. The EatA passenger domain is immunogenic and protective against ETEC infection of mice [23]. The *eatA* gene was present in ~70% of ETEC strains collected from Chile and Guinea Bissau [23].

Other proteins under investigation for their potential to be used in ETEC vaccines are the YghJ metalloprotease and EaeH, a putative adhesin [4]. YghJ degrades MUC2 and antibodies raised against YghJ inhibit LT delivery [24]. EaeH has immunoglobulin-like domains which are similar to domains of other proteins involved in adhesion [4]. Further studies are needed to realize the full potential of these proteins as vaccine candidates. Genes involved in the biogenesis of Type I fimbriae in ETEC are upregulated in their expression upon contact with epithelial cells [25]. Type I fimbriae were used as a vaccine candidate in pigs and significantly reduced the intestinal load of ETEC [26]. It may be possible to use highly conserved subunits of Type I fimbriae as potential vaccine candidates [4].

A major challenge to ETEC vaccine development lies in the genomic diversity of the strains [27]. Ultimately, it may be desirable to identify antigens that are sufficiently conserved to provide a broad range of protection against the diverse population of ETEC strains that cause diarrheal disease [4]. ETEC protective antigens remain relatively poorly defined in comparison with studies of other diarrheal pathogens. Indeed, with the exception of antibody responses to LT and CFs, relatively little is known about the human immune response to ETEC infection. Genome sequence data are now available for several ETEC isolates that have previously been studied in human vaccine trials. These data suggest that the genomes of these organisms are variable [27]. The apparent sequence diversity among strains suggests that identifying broadly conserved antigens with a “reverse vaccinology” approach [28], rather than focusing on a limited class of proteins (i.e. CFs) may be an effective strategy for vaccine development.

Bacterial surface proteins are attractive as targets as vaccine components [29]. However, identifying these proteins can be difficult, and in the past has typically required using chromatographic separation methods [30] susceptible to contamination [31]. ETEC utilizes a type II secretion system (T2SS) to transport proteins from the cytoplasm to the outer membrane [32]. While the substrates of the T2SS are incompletely characterized, we hypothesized that at least a subset of these proteins may be effective vaccine components. In the current study, we identified several ETEC proteins that differed in their abundance in membrane protein preparations from wild-type (WT) vs. a type II secretion mutant of ETEC. After purifying these proteins, we generated antisera and characterized the ability of these antisera to protect cultured intestinal epithelial cells from adherence by a diverse panel of ETEC strains differing in CF type. We also identified three ETEC proteins that provide protective immunity in an intranasal mouse challenge model.

## Methods

### Ethics statement

The Kansas State University Institutional Animal Care and Use Committee approved the animal procedures (IACUC protocol #3196) in the context of the Kansas State University Animal Welfare Assurance Number A3609-01, in compliance with the Public Health Service (PHS) Policy on Humane Care and Use of Laboratory Animals.

### Bacterial strains

The ETEC strains used are described in Table 1. ETEC H10407 was originally isolated from a patient with a severe cholera-like diarrhea in Bangladesh [33].

**Table 1:** ETEC strains used in this study.

Strain	CF type	Serotype	Location	Source/Reference
H10407	CFA/I	O78:H11	Bangladesh	[33]
H10407Δ*gspE*				[34]
H10407Δ*skp*				This study
H10407Δ*mipA*				This study
H10407Δ*2479*				This study
B7A	CS6	O148:H28	Vietnam	[35]
E24377A	CS1,CS3	O139:H28	Egypt	[36]
278485–1	CS2,CS3	O6:H16	Bangladesh	[37]
M421C1	CS5,CS6	SA:K83:H-	Morocco	[38]
2–1	CS6	O159:NM	Saudi Arabia	[39]
WS6866B	CS8	O25:H-	Egypt	[40]
2230	CS10	025:H16	Senegal	[41]
350C1A	CS12	O159:H4	Kenya	[38]
PE360	CS13	O9:H-	Australia	[42]
E7476A	CS14	O166:H27	South Africa	[43]
E20738A	CS17	O114:H21	Zaire	[44]
ARG-2	CS18	O20:K27:H-	Argentina	[45]
DS26-1	CS19	O8:H9	Saudi Arabia	[39]
WS2173A	CS23	O71:H4	Egypt	S. Savarino
WS4264A-1	CS25	O64:H-	Egypt	S. Savarino
WS7162A-1	CS27	O15:H40	Egypt	S. Savarino

The ETEC H10407 Δ*gspE* mutant was a gift from Dr. James M. Fleckenstein [21]. ETEC strains used for in vitro adherence assays were gifts from Dr. Stephen Savarino. Deletion mutants in the *skp*, *mipA*, and *ETEC_2479* genes were generated using the λ-Red recombinase method [46]. Mutants were confirmed by both PCR screening and DNA sequencing. ETEC were plated on colonization factor antigen (CFA) agar plates (1% Casamino acids, 0.15% yeast extract, 2% agar, 0.4 mM magnesium sulfate, 0.04 mM manganese chloride) and grown at 37°C. For adherence assays, individual colonies were inoculated in Luria-Bertani (LB) broth overnight followed by re-inoculation of overnight cultures in Eagle’s minimal essential medium (EMEM) to an OD_600_ of 0.6.

### Bacterial fractionation and mass spectrometry

Cytoplasmic and membrane protein fractions were obtained by growing WT and Δ*gspE* mutant ETEC H10407 strains overnight in 50 ml CAYE media (2% Casamino acids, 0.6% yeast extract, 43 mM NaCl, 38 mM K_2_HPO_4_, 0.25% glucose, 0.1% trace minerals). Bacterial cultures were centrifuged, washed in PBS, and then treated with lysozyme and recentrifuged. The pellet was resuspended in 10 mM Tris-HCl, pH 7.0, sonicated, and centrifuged. The supernatant was subjected to ultracentrifugation (1 h, 50,000 g, 4°C), after which the supernatant was retained as the cytoplasmic fraction. The pellet was resuspended in 10 mM Tris-HCl, pH 7.0 and retained as the membrane fraction. Equal amounts of protein from each fraction (~50 μg) were digested to peptides with trypsin, differentially labeled with light- and heavy-isotopologs of formaldehyde (Cambridge Isotope Labs), combined, resolved by isoelectric focusing, and analyzed by nLC-MS/MS using an LTQ-Orbitrap mass spectrometer. The Mascot search engine was used to compare the measured fragment spectra against an *in silico* translation of the ETEC H10407 genome.

### Antigen cloning, expression, and purification

PCR primers were designed so as to amplify genes without predicted signal-peptide coding sequences. PCR products were generated from chromosomal ETEC H10407 DNA and introduced into pET42a to generate recombinant proteins fused to a glutathione-*S*-transferase (GST) epitope. Plasmids were transformed into *E*. *coli* BL21(DE3) cells. Bacterial cultures were grown overnight at 37°C and subcultured 1:100 into fresh media. The subcultured cells were grown for 2 h at 37°C until reaching an OD_600_ of 0.2–0.5. Isopropyl β-D-1-thiogalactopyranoside (IPTG; 1 mM) was added and the bacterial cultures were grown for an additional 2 h, after which the cells were centrifuged (10 min, 10,000 g, 4°C). The cells were lysed in 1/20 culture volume of Bugbuster Protein Extraction Reagent (Novagen), rotated for 15 min at room temperature, and subjected to ultracentrifugation (1 h, 2,500 g, 4°C), after which the supernatant was retained. Supernatants were applied to GST-Bind Resin (Novagen), incubated at 4°C overnight with rotation, centrifuged (5 min, 1,000 g, 4°C), washed 3 times with GST Wash Buffer (Novagen), and eluted in GST elute buffer. The GST protein was also expressed separately and purified using similar conditions for use as an immunization control in subsequent experiments. The GST tags were not removed after protein purification. Potential contamination of antigen preparations with LPS was quantified using the Chromogenic Endotoxin Quantitation Kit (Pierce). LPS was present at negligible quantities in antigen preparations used for immunization (less than 3 pg/dose).

### Production of polyclonal antisera

Mice were immunized with 200 μg of each purified protein suspended in 50 mM Tris HCl pH 7.5, 50 mM NaCl, mixed with Complete Freund’s Adjuvant, and administered into multiple sites subcutaneously. Booster injections were administered twice at 2-week intervals, using 200 μg of antigen mixed with Incomplete Freund’s Adjuvant. Two weeks after the final immunization, the mice were euthanized, exsanguinated, and the blood was processed into serum. Control serum was also obtained from mice treated with PBS or a GST-epitope control protein. For each antigen, mouse sera were individually tested using ELISAs, then pooled by group, and stored at -80°C.

### Immunoassays

IgG and IgA concentrations in mouse sera and feces, respectively, were analyzed using ELISAs. We collected blood by intracardiac puncture after sacrificing the animals after challenge. To obtain fecal antibodies, we collected five fresh stool pellets from each animal. Stool pellets were added to 1 ml of fecal reconstitution buffer (50 mM ethylenediaminetetraacetic acid (EDTA), 0.1 mg/ml soybean trypsin inhibitor, 1.39 μg/ml phenylmethylsulfonylfluoride (PMSF), and homogenized. The samples were centrifuged (5 min, 5,000 g) to remove insoluble material and the supernatants were stored at -80°C.

ELISAs were performed in polystyrene 96-well, flat bottom plates (Whatman) coated with 0.5 μg/ml of each purified protein or BSA and incubated overnight at 4°C. Plates were washed 3X in PBS, 0.1% Tween-20 and blocked with 5% milk in PBS, 0.1% Tween-20 for 1 h at room temperature. For serum IgG analysis, 50 μl of each serum sample was added in duplicate to antigen-coated wells and incubated at 37°C for 1 h. Goat anti-mouse Ig (H+L) HRP detection antibody (Southern Biotech) diluted 1:4,000 in 0.1% PBS-Tween was added to the wells and incubated 37°C for 30 min. Plates were developed with 1-StepTM Ultra TMB-ELISA (Thermo) and quenched with 3 N H_2_SO_4_. Absorbance was read at 450 nm. Antibody titers were transformed logarithmically and the Student’s *t* test was used to compare the mean serum antibody titer values of different groups of mice with those of non-immunized mice. Differences in *P* values of < 0.05 were considered significant. For fecal IgA analysis, 50 μl of fecal supernatants were used with a rabbit anti-mouse IgA HRP detection antibody (Sigma) and developed as described above.

### In vitro adherence assays

HCT-8 cells (5*10^4^ cells/well) were grown in 24-well plates and incubated for 16 h in EMEM. We added serial dilutions (5 μg/ml to 500 pg/ml) of antisera prepared against ETEC antigens or control sera from non-immunized mice, followed by 5*10^5^ colony-forming units (CFUs) of ETEC strains that are diverse in CF-type (Table 1). HCT-8 cells were incubated with ETEC for 1 h at 37°C. Following incubation, cells were washed three times with sterile PBS to remove unbound ETEC and then lysed in 1% saponin. Cell lysates were serially diluted and plated for enumeration of adherent bacteria. We calculated the number of bacteria that remained cell-associated, relative to the bacterial inocula and computed the % reduction in adherence resulting from serum-containing polyclonal antibodies raised against ETEC proteins vs. serum from non-immunized mice. We compared the data statistically using paired Student’s *t* tests and considered p-values < 0.05 significant.

### Intranasal challenge assays

Female BALB/c mice (15/group) were obtained from the Jackson Laboratory (Bar Harbor, Maine). Mice were housed in microisolator cages and provided with food and water *ad libitum*. Antigens were administered intranasally at 20 μg/dose by mixing ETEC proteins with 2.5 μg of cholera toxin (Sigma-Aldrich) in 25 μl PBS to the external nares of mice lightly anesthetized with isoflurane. Booster doses were administered 2- and 4-weeks after the initial vaccination. Intranasal challenge studies were conducted as previously described [47, 48], with minor modifications. Two weeks after the final immunization, the mice were lightly anesthetized with isoflurane in a VetEquip RC2 isoflurane anesthesia machine and challenged intranasally with 5*10^8^ CFUs of ETEC H10407. To quantify changes to mouse clinical signs of illness as a function of ETEC challenge and immunization, we observed mice every 4 h after challenge and recorded the clinical signs of illness (lack of responsiveness to stimulation, hunched posture, ruffled hair coat, dehydration) as a function of time. Data were analyzed statistically using log-rank tests. If mice displayed clinical signs of illness, or at the end of the study (7 d), they were euthanized, necropsied, and their lungs were removed aseptically. Lungs were homogenized, serially diluted in PBS, and plated on MacConkey agar to enumerate ETEC.

## Results

### Antigen selection, purification, and serum IgG responses

We compared the relative abundance of proteins from membrane protein preparations between WT ETEC H10407 and an isogenic strain deficient in T2SS function (Δ*gspE*) using a mass spectrometry-based approach. GspE provides an ATP hydrolysis-dependent conformational change to the T2SS that drives protein secretion [49]. We isolated proteins from WT and Δ*gspE* ETEC and fractionated the bacterial proteins to cytoplasmic, secreted, and membrane fractions. We digested proteins with trypsin and differentially labeled the peptides with isotopologs of formaldehyde. We mixed equal quantities of protein from each sample and resolved them by in-solution isoelectric focusing (IEF). IEF fractions were further analyzed by nLC-MS/MS using an LTQ-Orbitrap mass spectrometer. We used the Mascot search engine to compare the measured fragment spectra against an *in silico* translation of the ETEC H10407 genome.

We computed the relative abundance ratios of the proteins that were isolated and identified from both WT and Δ*gspE* ETEC. We identified 402 proteins from 1,901 sequenced peptides and refined this list to 24 proteins that were enriched in the membrane fractions of WT, but not Δ*gspE* ETEC. Our analysis revealed several membrane proteins, flagellar subunits, and putative invasins (Table 2).

**Table 2:** ETEC H10407 proteins enriched in membrane protein preparations from WT vs. Δ*gspE*.

ETEC antigen	% AA identity to nearest *E*. *coli* K-12 match
CcmA, ATP-binding component of ABC transporter	100% over 205 AAs
ClpX, ATP-dependent Clp protease ATP-binding subunit	100% over 424 AAs
DLP12 prophage; truncated outer membrane porin	100% over 127 AAs
Dps, DNA starvation/stationary phase protection protein	100% over 167 AAs
EngA, GTP-binding protein	100% over 490 AAs
ETEC_2479, long-chain fatty acid outer membrane transporter	100% over 448 AAs
FabD, acyl carrier protein S-malonyltransferase	31% over 109 AAs
FlgE, flagellar hook protein	100% over 402 AAs
FliC, flagellin	52% over 543 AAs
MipA, MltA-interacting protein	99% over 248 AAs
LepA, GTP-binding protein	94% over 608 AAs
OmpA, outer membrane protein A	99% over 346 AAs
OmpR, osmolarity response regulator	38% over 239 AAs
OmpW, outer membrane protein W	100% over 212 AAs
OmpX, outer membrane protein X	100% over 171 AAs
Outer membrane porin protein C	100% over 367 AAs
Pbp5, penicillin-binding protein 5	100% over 403 AAs
Peptidyl-propyl cis-trans isomerase	32% over 146 AAs
Skp, periplasmic chaperone	99% over 161 AAs
Slp, outer membrane protein	99% over 188 AAs
SmpB, SsrA-binding protein	100% over 151 AAs
TibA, adhesin/invasin	33% over 942 AAs
TolC, outer membrane protein	100% over 493 AAs
UP12, universal stress protein UP12	100% over 142 AAs

**Fig 1:**
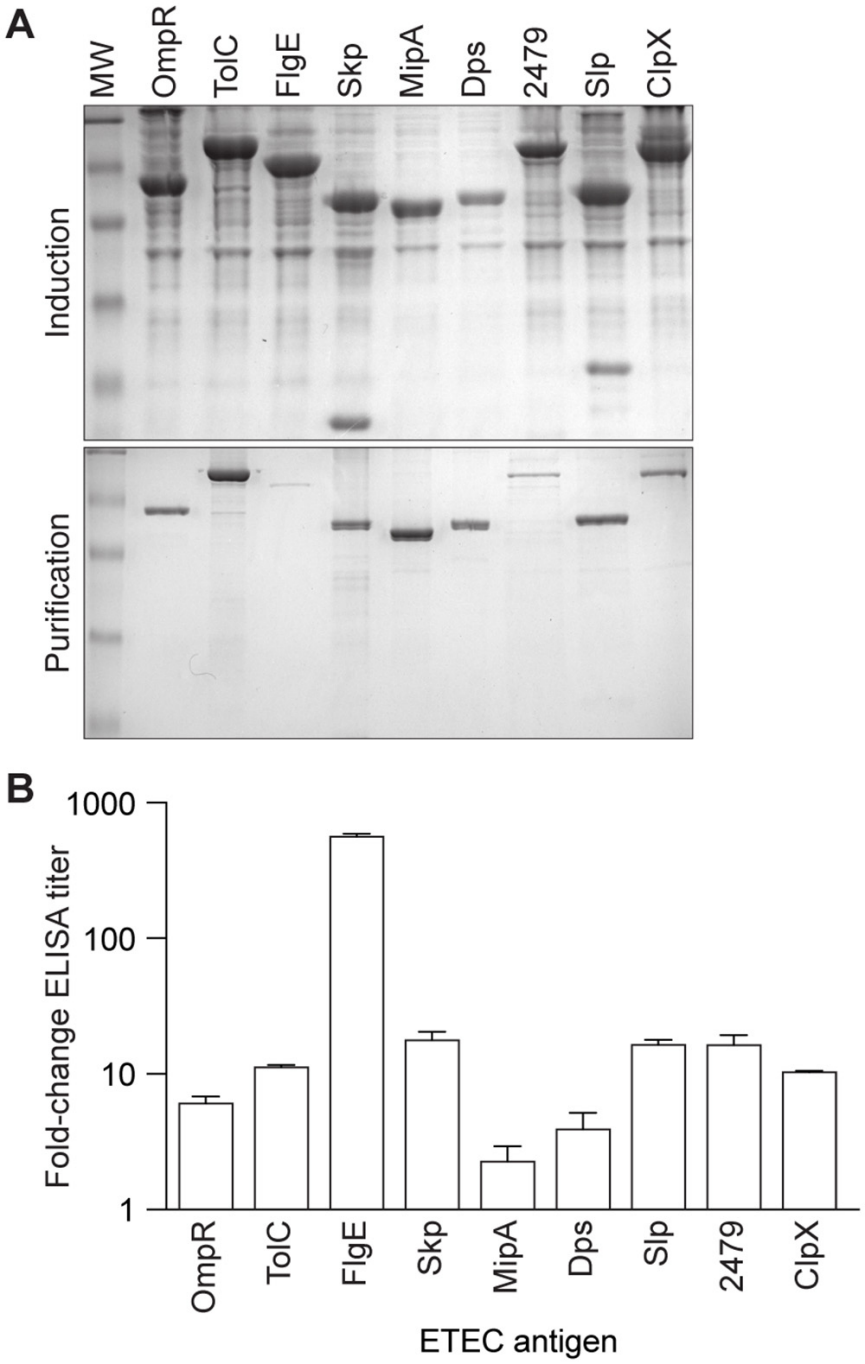
Antigen selection, purification, and serum IgG responses. **A.** Purification of ETEC antigens identified after bacterial fractionation and mass spectrometry. Top panel, induction in *E*. *coli* BL21 (DE3) cells; bottom panel, purification over glutathione *S*-transferase (GST) resin. **B.** Serum IgG responses in mice. Data are plotted as the fold-change in serum IgG after immunization with the indicated antigens, n = 3/group.

Of these 24 ETEC proteins identified, we cloned, expressed, and purified 9 antigens for further characterization, primarily based upon the solubility of recombinant forms of the proteins. We amplified the genes encoding the identified proteins in such a way to exclude predicted signal-peptides. PCR products were generated from chromosomal ETEC H10407 DNA and cloned as fusions to glutathione-S-transferase (GST). These plasmids were expressed in *E*. *coli* BL21(DE3) and GST-recombinant proteins were purified using GST-Bind Resin (Fig 1A). We used ELISAs to quantify antibody titers in pooled mouse sera. The IgG responses were variable, with the FlgE flagellar hook protein, yielding a 560-fold response (Fig 1B). Other antigens induced a 3- to 85-fold increase in IgG concentrations (Fig 1B). Several other proteins were not studied further in the context of this work, as they were partitioned into inclusion bodies and not easily maintained in a soluble form. It is conceivable that their fusion to other epitope tags or their purification in the presence of urea might overcome these challenges in future studies.

### Adherence assays

**Fig 2:**
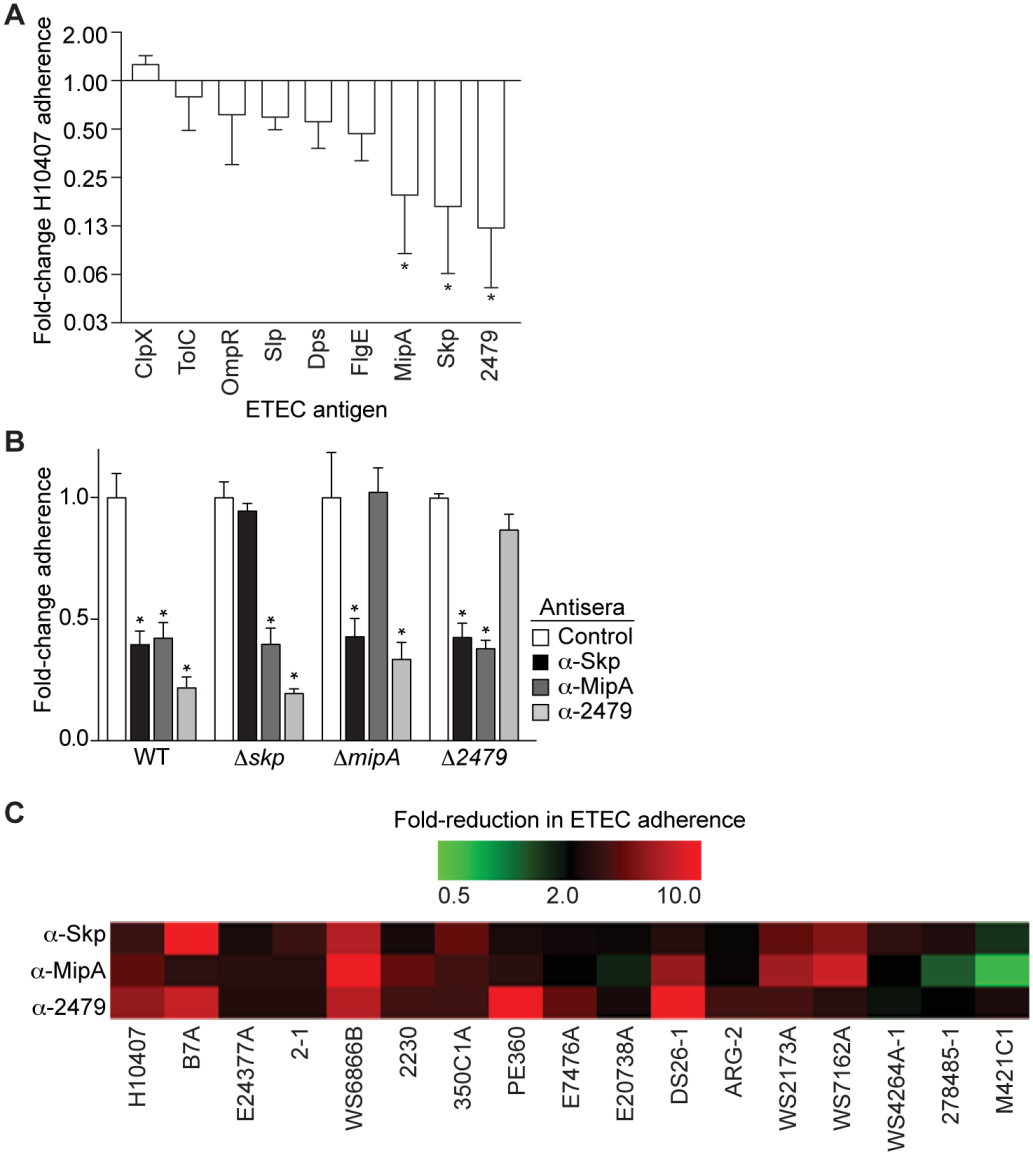
Adherence assays. **A.** Adherence of ETEC H10407 to HCT-8 cells in the presence of antisera. The fold-change in ETEC H10407 adherence is plotted as a function of incubation with the indicated antisera, after normalization to control samples. Asterisks indicate the antisera showing significant protection against ETEC H10407 that were selected for further characterization, n = 3/group. **B.** Analysis of antisera specificity. Deletion mutants were constructed for the *skp*, *mipA*, and *ETEC_2479* genes and then analyzed for their adherence to HCT-8 cells in the presence of the indicated antisera. Asterisks indicate significant reduction in bacterial adherence, n = 4-12/group. **C.** Heat map displaying the fold-change in adherence of the indicated ETEC strains to HCT-8 cells after their incubation with the indicated antisera. The scale ranges from 0.5 to a 10-fold decrease in adherence, as compared with control samples, n = 3/group. Red colors depict reduced ETEC adherence in the presence of antisera, while green colors depict increased adherence. The midpoint of the color gradient was set as a 2-fold reduction in adherence (black).

We quantified the extent to which the mouse antisera would subsequently protect against ETEC H10407 adherence to HCT-8 cells. Of the 9 antigens tested, 3 antigens (Skp, MipA, and ETEC_2479) showed a significant ability to inhibit H10407 adherence (Fig 2A). To determine the specificity of these antisera for their respective antigens, we deleted individually the genes encoding Skp, MipA, and ETEC_2479 and then re-evaluated the ability of the respective antisera to protect against the adherence of these ETEC mutants to HCT-8 cells. The antisera inhibited the WT strain and the 2 heterologous ETEC mutants, but no longer inhibited the adherence of the corresponding homologous ETEC mutant to as great an extent, suggesting that the phenotypes observed are largely attributable to antisera specificity (Fig 2B).

Antisera raised against Skp, MipA, and ETEC_2479 were further examined for their protective efficacy in reducing the adherence to HCT-8 cells of a panel of other ETEC strains that differ in CF-type (Table 1). Antisera raised against ETEC_2479 provided the highest degree of protection against the panel of ETEC strains. Antisera raised against Skp protected against most of the strains, except ETEC M421C1. Antisera raised against MipA protected against many strains, but failed to reduce the adherence of ETEC strains M421C1, 27845–1, and E20738A (Fig 2C).

### Intranasal challenges

**Fig 3:**
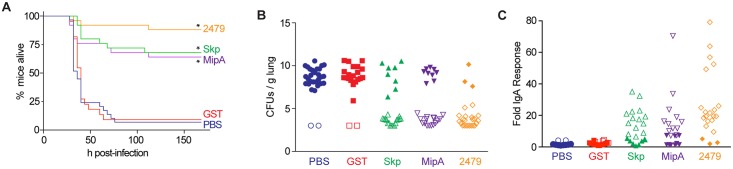
Intranasal challenge. **A.** Survival assay. The survival of mice is plotted as a function of time after mice were inoculated with ETEC H10407 following immunization with the indicated antigens. Asterisks indicate significant difference in survival, log-rank test, n = 22-29/group. **B.** ETEC loads (CFUs/g lung) in mice infected with ETEC H10407 at euthanasia or at the end of the study (7 d). Open symbols indicate mice that survived for the duration of the study. Closed symbols indicate mice that were euthanized due to their display of clinical signs of illness, n = 22-29/group. **C.** Fold change in mouse fecal IgA concentrations, n = 22-29/group, after inoculation with ETEC H10407 following immunization with the indicated antigens. Open symbols indicate mice that survived for the duration of the study. Closed symbols indicate mice that were euthanized due to their display of clinical signs of illness.

We conducted an intranasal challenge assay to evaluate the efficacy of immunizing mice against Skp, MipA, and ETEC_2479 in protecting mice against an otherwise lethal challenge with ETEC H10407. Mice (22-29/group) were immunized three times at two-week intervals with individual antigens combined with cholera toxin. Mice were inoculated intranasally with ETEC H10407 and then evaluated for clinical signs of disease over a 7-day period. All three antigens were protective against the infectious challenge to variable degrees (Fig 3A). ETEC_2479 was the most effective, providing protection for 22 of 25 (88%) of the immunized mice. Immunization with Skp or MipA yielded lesser, though still significant degrees of survival, with 17/25 (68%) and 16/25 (64%) of the mice surviving the infection. By contrast, only 2/29 (7%) of control mice treated with PBS and 2/20 (10%) of mice immunized with a GST purification control survived longer than 76 h after infection.

We observed high loads of ETEC (~10^9−10^ CFUs/g) in the lungs of mice that were euthanized due to their presentation of clinical signs of disease (Fig 3B). By contrast, relatively little ETEC (~10^3−5^ CFUs/g) was cultured from the lungs of mice that survived the infection (Fig 3B, compare open to closed symbols). ETEC loads were inversely related to the concentrations of IgA obtained from the feces of infected mice (Fig 3C). Fecal IgA responses were highly variable, but significantly correlated with mouse survival (Fig 3C, compare open to closed symbols). Mice that did not develop significant fecal IgA responses against the antigens did not survive the infectious challenge.

## Discussion

Bacterial surface proteins mediate adhesion and invasion to host cells and are often important vaccine candidates. The T2SS is considered important to ETEC virulence, as it is involved in secretion of proteins to the bacterial surface [50, 32]. We compared the difference in abundance between membrane protein preparations from ETEC H10407 strains possessing or lacking a functional T2SS system. *In vitro* adherence studies using the antisera produced in mice refined our study towards three antigens that showed a protective effect against bacterial adhesion to host cells. Similar approaches could be used to identify ETEC OMPs that are secreted by pathways other than the T2SS.

One limitation of our work is that many of the proteins we identified are somewhat unexpected and may not be entirely consistent with what might be expected from mutating the T2SS. The molecular basis for this result is unclear. However, we evaluated only the differential abundance of the proteins between the WT and the Δ*gspE* mutant and did not formally evaluate the secretion of these proteins by the T2SS. We rather chose to focus on the extent to which antibodies developed against these proteins might protect both against ETEC adherence in vitro and in the intranasal challenge model. Studying the mechanisms by which the ETEC antigens described are secreted may be interesting topics for future research.

The antigens identified in this study were effective in preventing ETEC attachment to HCT-8 cells, as well as in reducing lethality in an ETEC infectious challenge in mice. Skp is a molecular chaperone involved in outer membrane protein biogenesis that is believed to rescue misdirected OMPs [51] but may also function as a general chaperone [52]. The utility of using chaperones as vaccine candidates has been shown with other bacteria such as *Clostridium difficile* [53] and *Brucella melitensis* [54]. In addition, previous studies have suggested that Skp may be a component of the outer membrane [55]. MipA belongs to the MltA-interacting protein superfamily involved in remodeling peptidoglycan. Although few studies have focused on this protein as a vaccine candidate, it was previously identified as an ETEC immunoreactive protein [56]. MipA of *Salmonella paratyphi A* has been used as a vaccine candidate with moderate protection [57]. The ETEC_2479 long chain fatty acid transporter OMP is predicted to function as a multifunctional outer membrane porin essential for transport of long chain fatty acids and as a receptor for T2 bacteriophage [58]. Studies conducted in other Gram-negative bacteria have shown that porin proteins can be used as potential vaccine candidates [59].

Several other proteins identified in our initial proteomic analysis merit brief mention with regard to their potential use in vaccine studies. The OmpW protein is required for resistance to phagocytosis and is protective against intraperitoneal infectious challenge with *E*. *coli* [60]. ClpX was shown to have potential use in a vaccine to protect against the nasopharyngeal colonization of *Streptococcus pneumoniae* in mice [61]. OmpA is a major immunoreactive *E*. *coli* outer membrane protein that has been studied for its role in bacterial invasion of brain microvascular endothelial cells [62]. TibA is a 104 kDa outer membrane protein present on the *tib* locus of the ETEC chromosome that contributes to ETEC invasion [63] and shares similarity with several autotransporter adhesins of mucosal pathogens [4].

The three antigens we characterized in detail reduced the attachment of different ETEC strains to HCT-8 cells, independent of their CF-type, and might thus be useful in future vaccine preparations. The *in vivo* infectious challenge study with ETEC H10407 in mice showed that all the antigens were protective. Mice immunized with ETEC_2479 showed the best protection (88%), while mice immunized with Skp or MipA showed moderate protection (68 and 64%, respectively). Mice that survived the infectious ETEC challenge had higher fecal IgA concentrations, supporting the notion that the antigens elicited mucosal immune responses to prevent bacterial colonization. The inverse relationship of bacterial loads in the lungs, as well as the survival of mice, correlated well with the fecal IgA levels, as mice that survived the challenge had reduced ETEC loads, as compared with the mice that died.

Investigating ETEC intestinal colonization in mice requires pretreating the animals with antibiotics to remove other bacteria and the exact mechanism by which ETEC colonize the small intestine of mice is not fully understood [64]. One of the drawbacks of using the murine intestinal model for ETEC challenge is that mice do not develop diarrhea even when they are exposed to high doses of ETEC [21]. We were primarily interested in conducting survival assays to determine the efficacy of our vaccine candidates. We therefore elected to use an intranasal inoculation model. This model has been used previously as a model for enteric infections [47, 48]. The mouse intestinal epithelium shares similarity to the bronchus of the lung, as the lymphoid follicles present in the bronchial wall are similar to the Peyer’s patches of the intestine. Mucosal immunity is an important protective mechanism, as attachment to mucosal surfaces is often the first step in establishing infection. This protection is mainly provided by locally secreted sIgA, which neutralizes the pathogens at the point of infection [65]. If antigen is delivered at the mucosal surface, it can induce a strong immune response in the locally associated lymphoid tissue which, in turn, can travel to distal mucosal surfaces [66].

For vaccine development, it is important to target conserved antigens, yet, due to the diversity in ETEC serotypes and the large number of CFs identified, it is difficult to choose any one serotype-specific CF [67]. Finding broadly conserved, protective antigens is of primary importance. Our approach has resulted in finding three antigens that show heterologous protection against a variety of ETEC strains that are diverse in CF type. Preliminary analysis of a panel of 89 strains isolated from Bangladesh showed that MipA, Skp, and ETEC_2479 were prevalent in 89 (100%), 89 (100%), and 83 (93%) of the strains, respectively (D. Rasko, personal communication).

However, a potential drawback also exists, as these antigens are conserved among other pathogenic and commensal *E*. *coli* strains (Table 2), although the extent to which they are expressed and surface-exposed in these other strains is unclear. The potential impact of using a vaccine developed against antigens that are encoded by commensal organisms warrants further investigation. Future studies could conceivably quantify the extent to which vaccination with ETEC_2479, Skp, and MipA protect against other ETEC strains that have caused diarrheal disease in humans. Given the lack of full protection observed using each antigen independently, an additional plan for future work is also to evaluate the efficacy of a cocktail of the three antigens administered simultaneously.
